# In-Hospital Outcomes of Acute Kidney Injury After Pediatric Cardiac Surgery: A Meta-Analysis

**DOI:** 10.3389/fped.2021.733744

**Published:** 2021-09-03

**Authors:** Jef Van den Eynde, Hajar Rotbi, Marc Gewillig, Shelby Kutty, Karel Allegaert, Djalila Mekahli

**Affiliations:** ^1^Helen B. Taussig Heart Center, The Johns Hopkins Hospital and School of Medicine, Baltimore, MD, United States; ^2^Department of Cardiovascular Sciences, KU Leuven, Leuven, Belgium; ^3^Faculty of Medicine, Radboud University, Nijmegen, Netherlands; ^4^Department of Physiology, Radboud Institute for Health Sciences, Radboud University Medical Center, Nijmegen, Netherlands; ^5^Pediatric Cardiology, University Hospitals Leuven, Leuven, Belgium; ^6^Department of Development and Regeneration, KU Leuven, Leuven, Belgium; ^7^Department of Pharmacy and Pharmaceutical Sciences, KU Leuven, Leuven, Belgium; ^8^Department of Hospital Pharmacy, Erasmus Medical Center, Rotterdam, Netherlands; ^9^Department of Pediatric Nephrology, University Hospitals of Leuven, Leuven, Belgium; ^10^PKD Research Group, GPURE, Department of Development and Regeneration, KU Leuven, Leuven, Belgium

**Keywords:** acute kidney injury, cardiac surgery, children, meta-analysis, outcomes, pediatric cardiology, congenital heart disease, pediatric nephrology

## Abstract

**Background:** Cardiac surgery-associated acute kidney injury (CS-AKI) is associated with increased morbidity and mortality in both adults and children. This study aimed to investigate the in-hospital outcomes of CS-AKI in the pediatric population.

**Methods:** PubMed/MEDLINE, Embase, Scopus, and reference lists of relevant articles were searched for studies published by August 2020. Random-effects meta-analysis was performed, comparing in-hospital outcomes between patients who developed CS-AKI and those who did not.

**Results:** Fifty-eight publications between 2008 and 2020 consisting of 18,334 participants (AKI: 5,780; no AKI: 12,554) were included. Higher rates of in-hospital mortality (odds ratio [OR] 7.22, 95% confidence interval [CI] 5.27–9.88), need for renal replacement therapy (RRT) (OR 18.8, 95% CI 11.7–30.5), and cardiac arrhythmias (OR 2.67, 95% 1.86–4.80) were observed in patients with CS-AKI. Furthermore, patients with AKI had longer ventilation times (mean difference [MD] 1.76 days, 95% CI 1.05–2.47), pediatric intensive care unit (PICU) length of stay (MD 3.31, 95% CI 2.52–4.10), and hospital length of stay (MD 5.00, 95% CI 3.34–6.67).

**Conclusions:** CS-AKI in the pediatric population is associated with a higher risk of mortality, cardiac arrhythmias and need for RRT, as well as greater mechanical ventilation time, PICU and hospital length of stay. These results might help improve the clinical care protocols prior to cardiac surgery to minimize the disease burden of CS-AKI in children. Furthermore, etiology-specific approaches to AKI are warranted, as outcomes are likely impacted by the underlying cause.

## Introduction

Cardiac surgery-associated acute kidney injury (CS-AKI) is associated with increased morbidity and mortality (1). Although in-hospital outcomes of CS-AKI have been extensively studied in adults, those in children have not been subject to a systematic investigation. The renal physiology in children differs broadly from that in adults in terms of renal vasculature (2), maturity of the kidneys (2), and the impact of systemic processes such as inflammation (3) and coagulation (4). As a result, the mechanisms underpinning AKI development, the extent of recovery from AKI, and outcomes after AKI might be different in pediatric patients. Furthermore, AKI after pediatric cardiac surgery is common, occurring in 30–50% of procedures (5).

Several observational studies have investigated the outcomes of CS-AKI in children over the past few decades. In a Danish single-center cohort study of 1,128 consecutive children undergoing their first operation for congenital heart disease, Pedersen et al. (6) revealed that CS-AKI was independently associated with increased mortality. Subsequently, the prospective multicenter TRIBE-AKI study, including 311 children undergoing pediatric cardiac surgery, revealed an independent association of AKI with prolonged ventilation and increased length of stay (7). These findings have been confirmed in a recent secondary analysis of 799 patients in the Safe Pediatric Euglycemia after Cardiac Surgery trial (8).

In this systematic review and meta-analysis, our aim is to summarize the current knowledge regarding in-hospital outcomes of CS-AKI in the pediatric population.

## Materials and Methods

### Eligibility Criteria, Databases, and Search Strategy

We applied in our analysis two internationally validated protocols: PRISMA (9) and MOOSE (10). Studies were included if (i) the population consisted of pediatric patients (<18 years old), (ii) patients underwent cardiac surgery, (ii) in-hospital outcomes (any events occurring before hospital discharge) were compared between patients who developed AKI and those who did not, and (iv) they were prospective or retrospective observational studies or randomized controlled trials. Exclusion criteria were the following: (i) adult population, (ii) non-cardiac surgery, (iii) long-term outcomes, or (iv) data not available for AKI and no AKI separately.

Databases were searched for English language articles meeting the inclusion criteria and published by August 5, 2020: PubMed/MEDLINE, Embase, Scopus, and reference lists of relevant articles. The search terms for each of the databases were the following:

- PubMed (*n* = 1,656 on 6/08/2020): (acute renal failure OR acute kidney failure OR acute renal injury OR acute kidney injury OR acute renal insufficiency OR AKI OR acute renal dysfunction OR acute kidney dysfunction) AND (cardiac OR heart) AND (surgery OR operation OR pre-operative OR intraoperative OR perioperative) AND (pediatric OR neonate OR infant OR child OR adolescent) in all fields
- Embase (*n* = 1,275 on 6/08/2020): (‘acute renal failure'/exp OR ‘acute kidney failure’/exp OR ‘acute renal injury’/exp OR ‘acute kidney injury’/exp OR ‘acute renal insufficiency’/exp OR ‘aki’ OR ‘acute renal dysfunction’ OR ‘acute kidney dysfunction’) AND (‘cardiac’ OR ‘heart’/exp) AND (‘surgery’/exp OR ‘operation’/exp OR ‘preoperative’ OR ‘intraoperative’ OR ‘perioperative’) AND (‘pediatric’ OR ‘neonate’ OR ‘infant’ OR ‘child’ OR ‘adolescent’) in all fields
- Scopus (*n* = 1,256 on 6/08/2020): (TITLE-ABS-KEY (“acute renal failure” OR “acute kidney failure” OR “acute renal injury” OR “acute kidney injury” OR “acute renal insufficiency” OR “aki” OR “acute renal dysfunction” OR “acute kidney dysfunction”) AND TITLE-ABS-KEY (“cardiac” OR “heart”) AND TITLE-ABS-KEY (“surgery” OR “operation” OR “preoperative” OR “intraoperative” OR “perioperative”) AND TITLE-ABS-KEY (“pediatric” OR “neonate” OR “infant” OR “child” OR “adolescent”)

The following steps were taken: (1) identification of titles of records through databases searching, (2) removal of duplicates, (3) screening and selection of abstracts, (4) assessment of eligibility through full text articles, and (5) final inclusion in the study. Studies were selected by two independent reviewers (XJ and IP). When concordance was absent, a third reviewer took the decision to include or exclude the study (JVDE).

### Data Items

All outcomes that were reported by at least three studies were included in the meta-analysis. These outcomes included in-hospital mortality, need for renal replacement therapy (RRT), cardiac arrhythmias, ventilation time (days), pediatric intensive care unit (PICU) length of stay (days), and hospital length of stay (days). For studies reporting interquartile ranges, the mean was estimated according to a validated formula (11). Two independent reviewers extracted the data (BD and XJ). When concordance was absent, a third reviewer checked the data and took the final decision (JVDE). From each study, we extracted first authors' name, year of publication, country of origin, study design, years of enrollment, sample size, AKI incidence, definition of AKI, and mean age.

Three main definitions for AKI currently exist. First, the Risk for renal dysfunction, Injury to the kidney, Failure of kidney function, Loss of kidney function and End-stage renal disease (RIFLE) classification was published by the Acute Dialysis Quality Initiative in 2004 (12). Staging in this classification is based on changes from baseline serum creatinine (SCr) or glomerular filtration rate (GFR) within 7 days. This classification has been modified for children in the pediatric RIFLE (pRIFLE) classification, adding estimated creatinine clearance (eCrCl) (13). Secondly, another modification of the RIFLE classification has led to the AKI Network (AKIN) criteria, which focus on dynamic changes in creatinine (14). Contrary to RIFLE, AKIN does not use premorbid baseline SCr, but the lowest SCr within a 48-h period, as the reference SCr for calculations of absolute and relative increase in SCr values. The AKIN criteria also avoid the use of CrCl. The third and last modification is the Kidney Disease Improving Global Outcomes (KDIGO) classification, which covers both the AKIN and RIFLE criteria (15). This new classification combines the 1.5-fold relative increase in SCr and urine output over 7 days from the RIFLE criteria with the absolute increase in SCr of 0.30 mg/dL over the rolling 48-h window from AKIN. Urine output criteria are common to all three classifications.

Risk of bias of the selected studies was assessed using the Cochrane risk-of-bias tool for randomized trials (RoB 2) and the Risk Of Bias In Non-randomized Studies of Interventions (ROBINS-I), according to study design.

### Statistical Analysis

Odds ratios (OR) with 95% CI and *p*-values were calculated for binary variables. For continuous variables, mean differences (MD) with 95% confidence interval (CI) and *p*-values were considered. Chi-square test and *I*^2^ test were performed for assessment of statistical heterogeneity (16). The MD and OR were combined across the studies using two random-effects models: an inverse-variance method and a Mantel-Haenszel method, respectively (17). Funnel plots represent the analysis of publication bias, statistically analyzed by Begg and Mazumdar's rank correlation method (18) and Egger's linear regression method (19). In addition, the proportions of patients with AKI who died, required RRT, or developed cardiac arrhythmias were pooled.

Subgroup analyses were conducted based on the following predefined variables: definition of AKI (AKIN, KIGO, pRIFLE), age group defined based on the mean age of all participants in the individual study (neonates <1 month, infants 1–12 months, toddlers 12–36 months, and children >36 months, study design (retrospective, prospective), number of centers (single center, multicenter), and era (2001–2010, 2011–2020).

Meta-regression analyses were performed to determine whether the effect of AKI on in-hospital outcome was modulated by (1) AKI incidence (as a proxy of the overall risk profile of the study populations in the individual study) or (2) age. The regression coefficient hereby describes how the effect of AKI on outcome changes with a percentage increase in AKI incidence or a month increase in age, respectively. For binary risk factors, the log-transformed value of the effect of the risk factor was used in the regression model, and the exponential of the regression coefficient gives an estimate of the relative change in effect of the risk factor.

All analyses were completed with R Statistical Software (version 4.0.5, Foundation for Statistical Computing, Vienna, Austria).

## Results

### Study Selection and Characteristics

**Figure 1:**
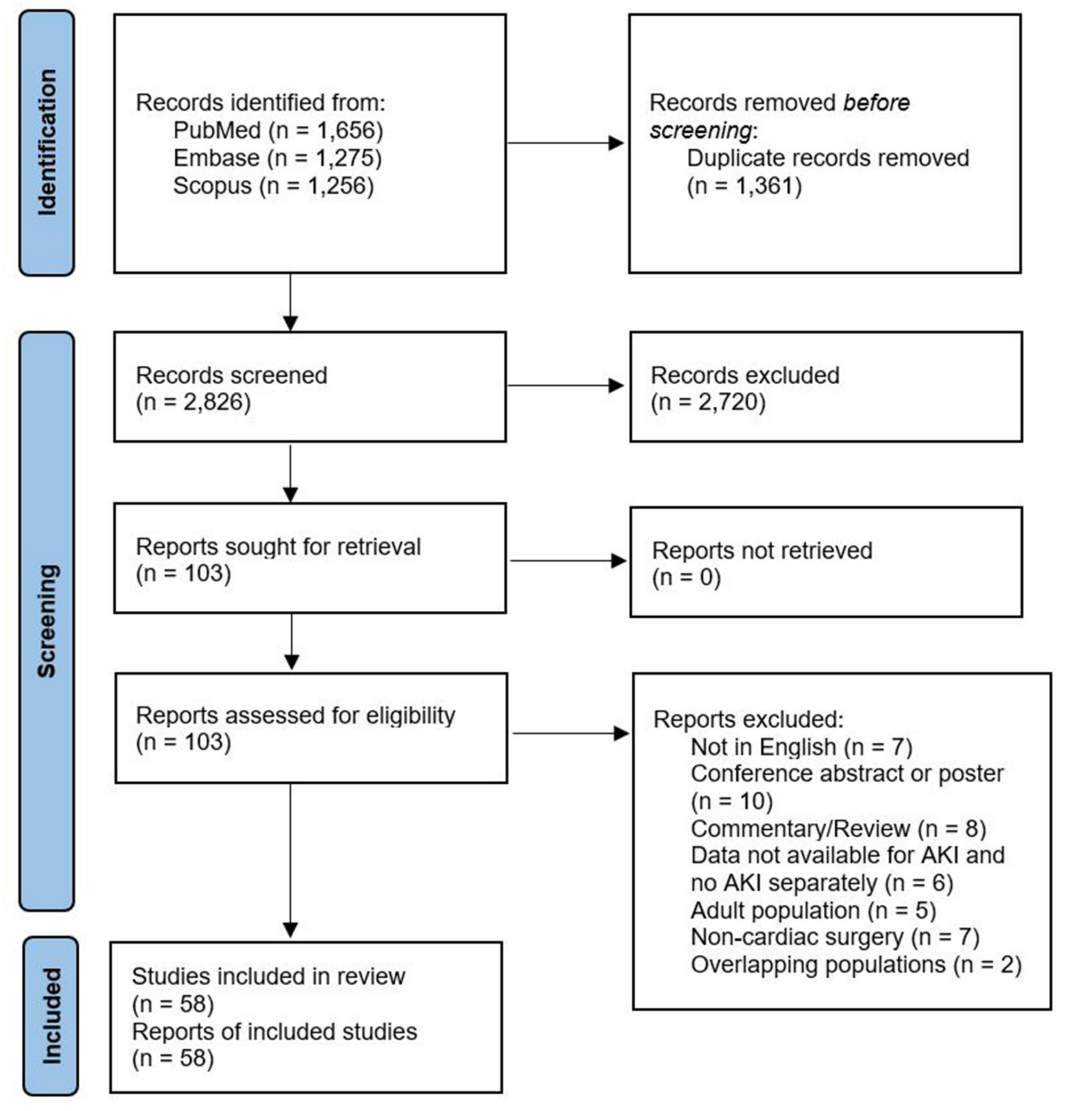
Flow diagram of studies included in data search.

A total of 2,826 citations were identified, of which 106 studies were potentially relevant and retrieved as full text. Fifty-eight publications from 2008 to 2020 fulfilled the eligibility criteria (Figure 1). Characteristics of each study and their participants are shown in Supplementary Material, Supplementary Table 1. A total of 18,334 participants (AKI: 5,780; no AKI: 12,554) were included. All studies were non-randomized observational studies, except for one randomized controlled trial (8). Of the former, 16 used a prospective design. The pooled mean age of participants was 25.7 months (58 studies, 18,334 participants). The pRIFLE criteria (13) were applied in 22 studies, while 15 studies used the KDIGO guidelines (15), 13 used the AKIN criteria (14), three used a combination of pRIFLE and AKIN, and three studies compared multiple definitions of AKI. In the two remaining studies AKI was detected based on doubling in SCr concentration from baseline or the need for acute dialysis during hospital stay. A total of 16 studies used urine output criteria in addition to the serum creatinine (SCr)-based criteria. The overall internal validity of the analysis was considered moderate risk of bias (Supplementary Material, Supplementary Figures 1–3).

### Synthesis of Results

#### Meta-Analysis

The results of the meta-analysis comparing in-hospital outcomes between pediatric patients who developed AKI following cardiac surgery and those who did not, are summarized in Table 1. The overall OR for in-hospital mortality indicated a significantly elevated risk in patients with AKI (random-effects model: OR 7.22, 95% CI 5.27–9.88, *p* < 0.001). There was evidence of high heterogeneity (*I*^2^ = 62%, *p* < 0.001) of the effect of AKI among the studies for in-hospital mortality. Similarly, a higher need for RRT was observed with AKI (random-effects model: OR 18.84, 95% CI 11.65–30.47, *p* < 0.001), with a high heterogeneity (*I*^2^ = 72%, *p* < 0.001). Furthermore, AKI was associated with an increased risk of cardiac arrhythmias (random-effects model: OR 2.67, 95% CI 1.86–4.80, *p* < 0.001). There was no evidence for significant heterogeneity with regards to this observation (*I*^2^ = 40%, *p* = 0.091). Proportion analysis revealed that of all pediatric patients who developed AKI following cardiac surgery, 9% (95% CI 6–11%) died during hospitalization, 8% (95% CI 5–12%) required RRT, and 29% (95% CI 23–36%) developed cardiac arrhythmias, although all with high heterogeneity (*I*^2^ = 90%, *I*^2^ = 93%, and *I*^2^ = 67%, all *p* < 0.001).

**Table 1:** Meta-analysis of in-hospital outcomes of AKI after pediatric cardiac surgery: summary of results.

**Outcome**	**Number of (sub)studies**	Effect size^*^	***p*-value**	***I*^2^**	***p*-value**
In-hospital mortality	49	7.22 (5.27–9.88)	** <0.001**	62%	<0.001
Need for RRT	50	18.84 (11.65–30.47)	** <0.001**	71%	<0.001
Cardiac arrhythmias	10	2.67 (1.86–4.80)	** <0.001**	40%	0.091
Ventilation time (days)	34	1.76 (1.05–2.47)	** <0.001**	99%	<0.001
PICU length of stay (days)	38	3.31 (2.52–4.10)	** <0.001**	97%	<0.001
Hospital length of stay (days)	36	5.00 (3.34–6.67)	** <0.001**	98%	<0.001

*AKI, acute kidney injury; PICU, pediatric intensive care unit; RRT, renal replacement therapy*.

* *Effect sizes are presented as odds ratios for binary outcomes and mean differences for continuous outcomes. Significant p-values (p < 0.05) are indicated in bold*.

Patients with AKI had longer ventilation times (random-effects model: MD 1.76 days, 95% CI 1.05–2.47, *p* < 0.001), longer PICU length of stay (random-effects model: MD 3.31 days, 95% CI 2.52–4.10, *p* < 0.001), and longer hospital length of stay (random-effects model: MD 5.00 days, 95% CI 3.34–6.67, *p* < 0.001). The heterogeneity was considerably high for all of these findings (all *I*^2^ ≥ 97%, *p* < 0.001).

Funnel plot analysis (Supplementary Material, Supplementary Figure 4) showed asymmetry around the axis for the need for RRT and ventilation time. Consequently, publication bias related to these outcomes cannot be excluded.

#### Outcomes in Neonates (<30 Days)

In an attempt to explain the observed heterogeneity in the reported outcomes, subanalyses were performed. These analyses revealed largely comparable results (Table 2). However, a significant interaction was observed of age group with the effect of AKI on in-hospital mortality, cardiac arrhythmias, and PICU length of stay. The OR for in-hospital mortality increased from 3.75 in neonates to 4.08 in infants, 9.29 in toddlers, and 11.80 in children (*p* = 0.004). Similarly, the OR for cardiac arrhythmias increased from no significant effect in neonates to 1.65 in infants, 3.16 in toddlers, and 5.49 in children (*p* < 0.001). Furthermore, AKI led to a greater increase in PICU length of stay in neonates (MD 3.57) and toddlers (MD 4.69) than in infants (MD 2.11) and children (MD 2.61) (*p* = 0.029).

**Table 2:** Results from the subanalyses.

		**Number of (sub)studies**	Effect size^*^	***I*^2^**	***p*-value**	**Interaction *p*-value**
**In-hospital mortality—effect of AKI on outcome**						
AKI definition	AKIN	13	10.00 (5.08; 19.69)	61%	0.002	0.237
	KDIGO	14	4.60 (2.28; 9.29)	47%	0.049	
	pRIFLE	19	7.49 (4.36; 12.86)	71%	<0.001	
Age group	Neonates	7	3.75 (1.80; 7.84)	0%	0.493	**0.004**
	Infants	11	4.08 (2.13; 7.84)	15%	0.304	
	Toddlers	20	9.29 (5.37; 16.10)	79%	<0.001	
	Children	11	11.80 (6.62; 21.02)	0%	0.628	
Study design	Retrospective	40	7.48 (5.29; 10.58)	57%	<0.001	0.493
	Prospective	9	5.65 (2.28; 14.02)	44%	0.098	
Number of centers	Single center	44	6.96 (4.93; 9.82)	66%	<0.001	0.414
	Multicenter	5	9.37 (3.83; 22.96)	0%	0.624	
Era	2001–2010	28	8.22 (5.66; 11.92)	55%	<0.001	0.218
	2011–2020	20	6.32 (3.29; 12.12)	60%	<0.001	
**In-hospital mortality—proportion analysis within AKI patients**						
AKI definition	AKIN	13	13.7% (8.4–21.6%)	82.7%	<0.001	0.089
	KDIGO	14	4.9% (2.4–9.6%)	60.7%	0.002	
	pRIFLE	19	7.9% (4.8–12.8%)	94.4%	<0.001	
Age group	Neonates	7	10.5% (7.0–15.3%)	48.5%	0.076	0.447
	Infants	11	5.8% (2.8–11.7%)	79.1%	<0.001	
	Toddlers	20	10.3% (6.8–15.3%)	94.0%	<0.001	
	Children	11	7.2% (2.9–16.6%)	81.2%	<0.001	
Study design	Retrospective	40	9.1% (6.5–12.6%)	90.8%	<0.001	0.360
	Prospective	9	6.4% (3.2–12.3%)	74.3%	<0.001	
Number of centers	Single center	44	8.9% (6.3–12.3%)	89.4%	<0.001	0.080
	Multicenter	5	6.1% (4.7–7.8%)	0%	0.587	
Era	2001–2010	28	8.5% (5.5–12.9%)	93.2%	<0.001	0.456
	2011–2020	20	8.3% (5.4–12.3%)	65.7%	0.001	
**Need for RRT—effect of AKI on outcome**						
AKI definition	AKIN	13	33.91 (13.55; 84.88)	81%	<0.001	0.256
	KDIGO	10	26.63 (4.21; 168.55)	64%	0.011	
	pRIFLE	23	13.17 (6.53; 26.56)	61%	<0.001	
Age group	Neonates	5	11.61 (2.31; 58.43)	59%	0.047	0.494
	Infants	9	14.28 (4.56; 44.77)	54%	0.032	
	Toddlers	18	29.84 (10.87; 81.93)	74%	<0.001	
	Children	17	13.20 (6.42; 27.17)	12%	0.320	
Study design	Retrospective	38	22.31 (12.50; 39.82)	77%	<0.001	**0.035**
	Prospective	11	8.64 (3.92; 19.03)	0%	0.590	
Number of centers	Single center	43	18.54 (10.69; 32.18)	74%	<0.001	0.659
	Multicenter	6	18.84 (11.65; 30.47)	0%	0.897	
Era	2001–2010	30	21.86 (10.45; 45.75)	80%	<0.001	0.277
	2011–2020	19	13.43 (7.58; 23.77)	4%	<0.001	
**Need for RRT—proportion analysis within AKI patients**						
AKI definition	AKIN	13	13.0% (6.0–26.2%)	95.7%	<0.001	0.166
	KDIGO	10	5.4% (2.0–14.1%)	79.0%	<0.001	
	pRIFLE	23	8.0% (4.6–13.8%)	91.1%	<0.001	
Age group	Neonates	5	26.2% (11.0–50.5%)	96.7%	<0.001	**0.050**
	Infants	9	8.0% (3.3–18.0%)	89.0%	<0.001	
	Toddlers	18	8.2% (4.7–14.1%)	94.5%	<0.001	
	Children	17	5.1% (2.2–11.2%)	63.1%	0.012	
Study design	Retrospective	38	8.7% (5.4–13.8%)	93.5%	<0.001	0.359
	Prospective	11	6.2% (3.6–10.6%)	62.4%	0.014	
Number of centers	Single center	43	8.1% (5.1–12.7%)	92.5%	<0.001	0.548
	Multicenter	6	6.5% (3.6–11.5%)	86.1%	<0.001	
Era	2001–2010	30	7.1% (3.8–12.9%)	94.3%	<0.001	0.507
	2011–2020	19	9.2% (5.9–13.9%)	84.0%	<0.001	
**Cardiac arrhythmias—effect of AKI on outcome**						
AKI definition	AKIN	3	3.51 (0.57; 21.53)	46%	0.159	0.149
	KDIGO	4	1.69 (0.99; 2.87)	39%	0.457	
	pRIFLE	2	3.61 (0.28; 46.25)	0%	0.415	
Age group	Neonates	2	2.39 (0.00; 1822.33)	52%	0.150	** <0.001**
	Infants	3	1.65 (1.01; 2.69)	0%	0.672	
	Toddlers	2	3.16 (1.54; 6.50)	0%	0.749	
	Children	3	5.49 (1.60; 18.88)	0%	0.581	
Study design	Retrospective	10	2.67 (1.86–4.80)	40%	0.091	N/A
	Prospective	0	N/A	N/A	N/A	
Number of centers	Single center	10	2.67 (1.86–4.80)	40%	0.091	N/A
	Multicenter	0	N/A	N/A	N/A	
Era	2001–2010	7	3.01 (2.01; 4.51)	11%	0.344	0.286
	2011–2020	3	2.11 (0.60; 7.38)	43%	0.172	
**Cardiac arrhythmias—proportion analysis within AKI patients**						
AKI definition	AKIN	3	25.8% (16.7–37.7%)	75.7%	0.032	0.724
	KDIGO	4	32.0% (19.3–48.0%)	71.4%	0.024	
	pRIFLE	2	28.3% (18.0–41.6%)	83.5%	0.021	
Age group	Neonates	2	42.6% (30.9–55.2%)	74.9%	0.031	**0.017**
	Infants	3	22.7% (16.6–30.1%)	0%	0.583	
	Toddlers	2	35.3% (29.0–42.2%)	0%	0.632	
	Children	3	27.6% (15.1–45.0%)	74.9%	0.014	
Study design	Retrospective	10	29.0% (23.1–35.7%)	66.6%	0.091	N/A
	Prospective	0	N/A	N/A	N/A	
Number of centers	Single center	10	29.0% (23.1–35.7%)	66.6%	0.091	N/A
	Multicenter	0	N/A	N/A	N/A	
Era	2001–2010	7	29.7% (22.3–38.2%)	69.8%	0.051	0.781
	2011–2020	3	27.8% (18.8–39.1%)	71.5%	0.034	
**Ventilation (days)**						
AKI definition	AKIN	8	1.95 (0.00; 3.91)	99%	<0.001	0.125
	KDIGO	11	0.97 (0.03; 1.90)	99%	<0.001	
	pRIFLE	15	2.39 (1.12; 3.67)	99%	<0.001	
Age group	Neonates	6	1.76 (0.17; 3.35)	97%	<0.001	0.230
	Infants	6	1.47 (−0.35; 3.29)	99%	<0.001	
	Toddlers	14	2.50 (0.90; 4.10)	99%	<0.001	
	Children	8	0.98 (0.26; 1.71)	99%	<0.001	
Study design	Retrospective	25	1.98 (1.09; 2.86)	99%	<0.001	0.351
	Prospective	9	1.27 (−0.19; 2.72)	99%	<0.001	
Number of centers	Single center	33	1.70 (0.99; 2.41)	99%	<0.001	**0.002**
	Multicenter	1	4.78 (2.96; 6.60)	N/A	N/A	
Era	2001–2010	16	1.68 (0.72; 2.65)	99%	<0.001	0.795
	2011–2020	18	1.87 (0.72; 3.02)	98%	<0.001	
**PICU length of stay (days)**						
AKI definition	AKIN	9	2.78 (2.20; 3.36)	96%	<0.001	0.156
	KDIGO	11	2.62 (1.46; 3.77)	95%	<0.001	
	pRIFLE	14	4.11 (2.14; 6.07)	98%	<0.001	
Age group	Neonates	5	3.57 (2.68; 4.46)	93%	<0.001	**0.029**
	Infants	8	2.11 (0.69; 3.52)	96%	<0.001	
	Toddlers	13	4.69 (2.63; 6.74)	98%	<0.001	
	Children	12	2.61 (1.67; 3.55)	94%	<0.001	
Study design	Retrospective	16	3.45 (2.39; 4.50)	97%	<0.001	0.525
	Prospective	12	2.97 (1.76; 4.18)	97%	<0.001	
Number of centers	Single center	33	3.34 (2.44; 4.25)	97%	<0.001	0.953
	Multicenter	5	3.29 (1.06; 5.52)	97%	<0.001	
Era	2001–2010	22	3.16 (2.11; 4.21)	98%	<0.001	0.225
	2011–2020	15	3.27 (2.14; 4.41)	96%	<0.001	
**Hospital length of stay (days)**						
AKI definition	AKIN	9	5.27 (3.00; 7.54)	97%	<0.001	0.081
	KDIGO	12	2.87 (0.73; 5.00)	98%	<0.001	
	pRIFLE	11	7.91 (3.15; 12.67)	97%	<0.001	
Age group	Neonates	6	4.73 (−2.52; 11.99)	97%	<0.001	0.516
	Infants	9	3.56 (0.52; 6.60)	98%	<0.001	
	Toddlers	10	7.47 (2.32; 12.63)	98%	<0.001	
	Children	11	4.94 (2.65; 7.23)	98%	<0.001	
Study design	Retrospective	27	4.51 (2.44; 6.68)	98%	<0.001	0.255
	Prospective	9	6.49 (3.23; 9.75)	98%	<0.001	
Number of centers	Single center	31	4.72 (2.83; 6.61)	98%	<0.001	0.325
	Multicenter	5	6.28 (2.70; 9.86)	96%	<0.001	
Era	2001–2010	23	4.20 (2.35; 6.04)	99%	<0.001	0.135
	2011–2020	13	7.12 (3.32; 10.92)	96%	<0.001	

*AKI, acute kindey injury; AKIN, Acute Kidney Injury Network; KDIGO, Kidney Disease Improving Global Outcomes; PICU, pediatric intensive care unit; pRIFLE, pediatric RIFLE; R, randomized; RIFLE, Risk for renal dysfunction, Injury to the kidney, Failure of kidney function, Loss of kidney function and End-stage renal disease; RRT, renal replacement therapy. Significant p-values (p <0.05) are indicated in bold*.

Age group did not affect the proportion of AKI patients who died (*p* = 0.447), although differences were observed with regard to the proportion of need for RRT and cardiac arrhythmias among AKI patients. The need for RRT was most common in neonates (26.2%), compared to 8.0% in infants, 8.2% in toddlers, and 5.1% in children (*p* = 0.050). Similarly, cardiac arrhythmias occurred in most commonly in neonates (42.6%) compared to 22.7% in infants, 35.3% in toddlers, and 27.6% in children (*p* = 0.017).

#### Other Subgroup Analyses

With regard to need for RRT, retrospective studies reported a significantly higher OR than prospective studies (22.31 vs. 8.64, *p* = 0.035). One multicenter study reported a significantly larger increase in ventilation time resulting from AKI when compared to single center studies (*p* = 0.002). No other significant interaction effects were observed and none of the outcomes differed according to AKI definition.

#### Meta-Regression

The results of the meta-regression analyses of outcomes as a function of AKI incidence (as a proxy for risk profile of the population) and age are summarized in Table 3. A significant association between AKI incidence and need for RRT was found: for each percentage increase in AKI incidence, the effect size of AKI on the need for RRT decreased by 3% (OR 0.970, 95% CI 0.944–0.997, *p* = 0.028). In addition, a significant association was found between age and cardiac arrhythmias: for each month increase in age, the effect size of AKI on cardiac arrhythmias increased by 2.7% (OR 1.027, 95% CI 1.005–1.050, *p* = 0.023). No significant associations were observed for other in-hospital outcomes as function of AKI incidence and age.

**Table 3:** Meta-regression of outcomes of AKI after pediatric cardiac surgery as a function of AKI incidence and age: summary of results.

**Outcome**	**Number of (sub)studies**	Regression coefficient^*^	Exp (regression coefficient)^**^	***t*-value**	***p*-value**
**As a function of AKI incidence**				
In-hospital mortality—effect of AKI on outcome	49	−0.010 (−0.031; 0.011)	0.990 (0.969; 1.011)	−0.962	0.342
In-hospital mortality—proportion analysis within AKI patients	49	−0.009 (−0.028; 0.011)	0.991 (0.972; 1.011)	−0.854	0.393
Need for RRT—effect of AKI on outcome	50	−0.030 (−0.057; −0.003)	0.970 (0.944; 0.997)	−2.277	**0.028**
Need for RRT—proportion analysis within AKI patients	50	−0.003 (−0.028; 0.022)	0.997 (0.972; 1.022)	−0.216	0.829
Cardiac arrhythmias—effect of AKI on outcome	10	0.012 (−0.019; 0.043)	1.012 (0.981; 1.044)	0.873	0.408
Cardiac arrhythmias—proportion analysis within AKI patients	10	−0.017 (−0.034; 0.001)	0.983 (0.967; 1.001)	−2.029	0.052
Ventilation time (days)	34	0.023 (−0.018; 0.064)	/	1.143	0.262
PICU length of stay (days)	38	0.003 (−0.043; 0.049)	/	0.141	0.889
Hospital length of stay (days)	36	0.032 (−0.073; 0.137)	/	0.622	0.538
**As a function of age**				
In-hospital mortality—effect of AKI on outcome	49	0.010 (−0.003; 0.024)	1.010 (0.997; 1.024)	1.529	0.134
In-hospital mortality—proportion analysis within AKI patients	49	0.012 (−0.003; 0.026)	1.012 (0.997; 1.026)	1.554	0.120
Need for RRT—effect of AKI on outcome	50	0.010 (−0.008; 0.032)	1.012 (0.992; 1.033)	1.233	0.225
Need for RRT—proportion analysis within AKI patients	50	−0.009 (−0.027; 0.008)	0.991 (0.973; 1.008)	−1.062	0.288
Cardiac arrhythmias—effect of AKI on outcome	10	0.027 (0.005; 0.049)	1.027 (1.005; 1.050)	2.801	**0.023**
Cardiac arrhythmias—proportion analysis within AKI patients	10	−0.001 (−0.020; 0.017)	0.999 (0.980; 1.017)	−0.114	0.909
Ventilation time (days)	34	−0.009 (−0.049; 0.030)	/	−0.474	0.639
PICU length of stay (days)	38	−0.001 (−0.037; 0.035)	/	−0.073	0.942
Hospital length of stay (days)	36	0.049 (−0.045; 0.144)	/	1.064	0.295

*AKI, acute kidney injury; PICU, pediatric intensive care unit; RRT, renal replacement therapy*.

* *The regression coefficient describes how the effect of AKI on the outcome changes with a percentage increase in AKI incidence (as a proxy of the overall risk of the population)*.

** *For binary risk factors, the log-transformed value of the effect of AKI on the outcome was used in the regression model, and the exponential of the regression coefficient gives an estimate of the relative change in effect of AKI on the outcome with a percentage increase in AKI incidence. Significant p-values (p < 0.05) are indicated in bold*.

## Discussion

### Summary of Evidence

**Figure 2:**
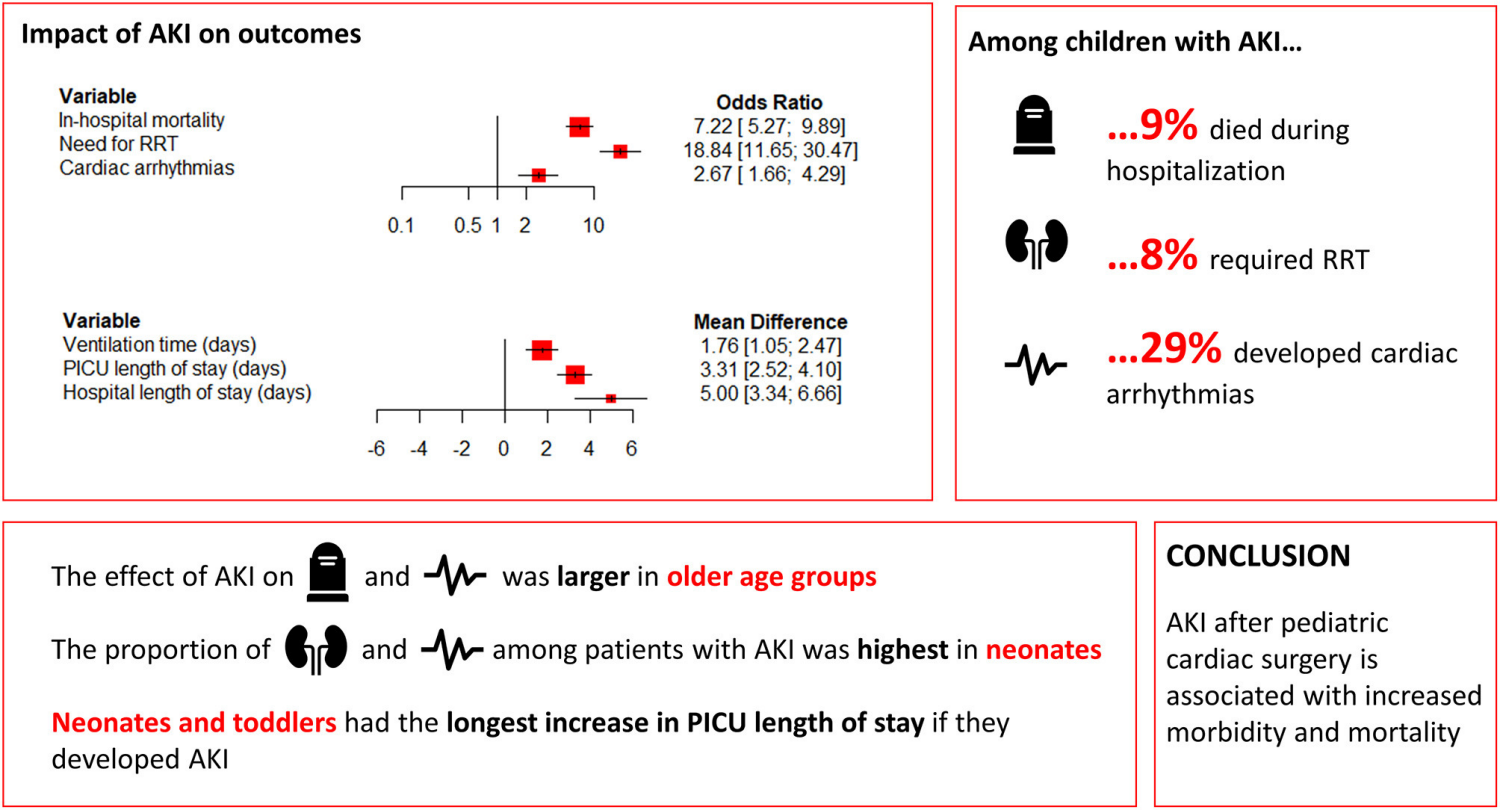
Forest plot summarizing main findings. AKI, acute kidney injury; PICU, pediatric intensive care unit; RRT, renal replacement therapy.

The present meta-analysis, including 18,334 participants from 58 studies, identified in-hospital outcomes that were significantly associated with AKI in pediatric patients following cardiac surgery. The main findings of our study are summarized in Figure 2. Higher rates of in-hospital mortality, cardiac arrhythmias, and need for RRT were observed in patients with post-operative AKI. Patients with AKI had longer ventilation times, longer PICU length of stay, and longer hospital length of stay. Subanalyses revealed largely comparable results, although the effect of AKI on in-hospital mortality and cardiac arrhythmias was larger in older age groups. Nonetheless, age group did not affect the proportion of AKI patients who died, while need for RRT and the occurrence of cardiac arrhythmias were most common in neonates. Neonates and toddlers had the longest increase in PICU length of stay if they developed AKI. Meta-regression confirmed the relationship between older age and increasing impact of AKI on cardiac arrhythmias, and revealed that the effect of AKI on the need for RRT decreased with increasing AKI incidence. Lastly, the findings of this meta-analysis highlight that an etiology-specific approach to AKI is warranted, as outcomes are likely impacted by the underlying cause.

### In-Hospital Mortality

Our proportion analysis revealed that of all children who developed AKI following cardiac surgery, 9.0% died during hospitalization. This is lower than the 15.3% reported by Sutherland et al. (20) in a cross-sectional analysis of 10,322 children who experienced in-hospital AKI in the 2009 Kids Inpatient Database. Subgroup analysis of their data revealed that mortality was even higher if AKI occurred in the ICU (32.8%), in neonates (31.2%), or when RRT was required (27.1%). Although age-based subgroup analyses were conducted in the present meta-analysis, no significant differences in the rates of in-hospital mortality was found between neonates and older children. While Sutherland and colleagues presented an analysis of outcomes after AKI regardless of etiology, the literature suggests that great differences in mortality rates exist between populations. For example a multicenter study by Fitzgerald et al. (21), which focused on children with severe sepsis, reported an in-hospital mortality rate of 52% in patients with AKI. In contrast, outcomes after AKI caused by hemolytic uremic syndrome (HUS) have greatly improved over the past decades, with a low death rate of 1–4% of patients in most reports (22). These findings highlight the benefit of an etiological approach to AKI, as risk factors, underlying mechanisms, modifying factors, and outcomes might greatly differ.

While no significant differences in the proportion of in-hospital mortality were seen between age groups, AKI was associated with a greater increase in the risk of mortality in older children (OR 11.80) compared to neonates (OR 3.75). There might be several explanations for this observation. Most of the included studies used SCr-based criteria in order to define AKI. Because neonates tend to have higher SCr values at baseline (23, 24), it is problematic that AKIN and KDIGO consider both a ≥0.3 mg/dL increase and a 1.5-fold increase in SCr as AKI. For a neonate with a baseline SCr of 1.2 mg/dL, AKI would already be diagnosed based on a post-operative SCr of 1.5 mg/dL (0.3 mg/dL increase) whereas the 1.5-fold increase criterion would only be met if a SCr of 1.8 mg/dL is observed. AKI criteria might have been more sensitive in neonates, thus more readily detecting patients with trivial changes in renal function which are not likely to affect the risk of mortality. This might also in part explain why the proportion of in-hospital mortality among neonates was considerably lower than that reported by Sutherland et al. (20), who used International Classification of Diseases, Ninth Revision, Clinical Modification (ICD-9-CM) codes to define AKI. Another possible explanation is that other mechanisms leading to mortality, apart from AKI, might play an important role in neonates. It has been demonstrated that inflammatory and coagulopathic complications of cardiac surgery are more pronounced in neonates and young infants (25, 26).

While this could not be investigated in the present meta-analysis, the Assessment of Worldwide Acute Kidney Injury, Renal Angina, and Epidemiology (AWARE) study (27) in critically ill children has previously demonstrated that a stepwise increase in the maximum stage of AKI (based on the KDIGO criteria) conferred an incremental risk of death. In addition, RRT and use of vasoactive support were found to have the strongest association with mortality. Of interest, the recently published Neonatal and Pediatric Heart and Renal Outcomes Network (NEPHRON) study reported outcomes of CS-AKI (also based on the KDIGO criteria) in 2,240 neonates. This study found that only stage 3 CS-AKI was associated with in-hospital mortality. The investigators therefore concluded that the KDIGO criteria may not be ideally suited to define a clinically meaningful renal injury phenotype in neonates with congenital heart disease. These findings are in line with the present meta-analysis, where the association of CS-AKI with in-hospital mortality was rather modest in neonates and more pronounced in older children.

### Need for RRT

The proportion of need for RRT in patients who developed AKI (8.0%) found in this meta-analysis is comparable to those reported by Sutherland et al. (20) (8.8%). Similarly, the AWARE study reported need for RRT in 5.1% of all patients, which increased with a stepwise increase in the maximum stage of AKI (27). Interestingly, we found that the effect size (in terms of OR) of AKI on the need for RRT decreased by 3% for each percentage increase in AKI incidence. Populations with a higher AKI incidence are assumed to have a higher baseline risk of developing renal complications—thus, more likely both to develop AKI and to require RRT. Factors other than AKI might thus become relatively more important drivers of the need for RRT in populations with high incidence of AKI. Another explanation might be that lower AKI incidence indicates more strict definition of AKI, where more severe forms of renal injury better predict the need for RRT.

### Cardiac Arrhythmias

A significantly elevated risk for arrhythmias in patients with AKI was found (OR = 2.67), and these were most frequently seen in neonates (42.6%). The predominant cause was electrolyte imbalances caused by AKI, especially hyperkalemia and hypocalcemia (28). These imbalances result in changes in cardiac ionic currents kinetics and are known precipitators and facilitators of potentially lethal ventricular arrhythmias. In addition, distorted intracellular calcium homeostasis due to changes in the dihydropyridine receptor on the L-type calcium channels of cardiomyocytes has been demonstrated in preclinical models of AKI (29). Our subanalyses and meta-regression revealed that the association of AKI with cardiac arrhythmias increased with age. This may be related to the higher baseline risk for arrhythmia in younger children, as evidenced by younger age at pediatric cardiac surgery being an independent risk factor of arrhythmias in previous investigations (30). The relative increase in risk of arrhythmias with AKI (expressed as OR) thus seems to be smaller in younger children, suggesting that the relative contribution of AKI to arrhythmia risk becomes more important with increasing age.

### Length of Stay

We found that patients with AKI had longer hospital length of stay, which is in line with the findings of Sutherland et al. (20). In their study, a median length of stay of 9 days was observed in pediatric patients with AKI, compared to 2 days in those without AKI (*p* < 0.001). They also reported longer length of stay among neonates with AKI compared to other age groups (median: 29 vs. 7 days, *p* < 0.001). Similar results were found in our subgroup analysis. AKI may lead to longer need for hospitalization in younger patients because of their small and immature kidneys with limited possibilities to compensate for renal ischemia, specific comorbidities, severity of the underlying cardiopathy, and surgery which is usually longer and more complex (2). Notably, data about the care processes that take place during this prolonged hospitalization are scarce. It is possible that patients stay in the hospital for diagnostic and/or therapeutic procedures related to the consequences of AKI. Several studies have reported increased need for ICU supplies, laboratory, pharmacy, respiratory devices, and dialysis associated with AKI, all of which have high costs (31, 32). Nonetheless, it cannot be excluded that reverse causality might be at play: patients who stay in hospital and ICU settings for a long time are more likely to develop hypotensive episodes and renal complications, among others due to the exposure to antihypertensives and nephrotoxic drugs (33).

### Perspectives for Future Research and Clinical Practice

Future research and clinical practice should focus on early detection of CS-AKI in pediatric populations, in order to avert the detrimental consequences of progressive renal injury and functional decline. For example, close monitoring of electrolyte disturbances and timely intervention, such as early correction of hyperkalemia, can reduce the risk of cardiac arrhythmias. More conservative use of inotropes may be advisable (34). Furthermore, several strategies to prevent AKI could be applied in the perioperative setting, including remote ischemic preconditioning (35), aminophylline (36), dexmedetomidine (36), acetaminophen (36), corticosteroids (36), and fenoldopam (36). The efficacy of these therapies not yet been demonstrated consistently, and the optimal protocol will likely require the combination of various strategies. The use of so-called “care bundles” in the treatment of AKI has been linked to reduced adverse outcomes in adults (37). These are designed to be a structured method of improving care processes and outcomes. One example is the SHOUT (“sepsis,” “hypovolaemia,” “obstruction,” “urinalysis,” and “toxins”) which defines the immediate response on detection of an AKI episode (38). Another initiative is Baby NINJA (Nephrotoxic Injury Negated by Just-in-Time Action) (39). Implementation of such “care bundles” adapted to pediatric cardiac surgery might hold promise for the future. Lastly, novel sensitive biomarkers could help with the identification of tubular injury in early stages and to intervene timely (40).

### Limitations and Sources of Heterogeneity

Several limitations need to be considered when interpreting the results. First, our study reported crude ORs, as most of the studies did not report adjusted ORs for the investigated outcomes; residual confounding in our pooled estimations is not unlikely. Nonetheless, we investigated potential confounders using subgroup and meta-regression analyses. Second, the interpretation of the results is complicated by statistical and clinical heterogeneity. Congenital heart disease is a diverse entity, and patients might present with varied physiology, surgical risk, and predisposition to adverse outcomes. The use of multiple definitions of AKI might be another reason for heterogeneity, although no significant differences could be observed between AKIN, KDIGO, and pRIFLE for any of the outcomes. Other sources of heterogeneity might include study design, age, numbers of centers, and era. Although subgroup analysis to some extent decreased the heterogeneity, it was insufficient due to the lack of raw data. Similarly, meta-regression analysis did not identify the exact causes responsible for heterogeneity. These first two limitations were also reflected by the risk of bias which was assessed as moderate, mostly because of bias due to confounding and bias in measurement of outcome. Third, our analysis was limited to those in-hospital outcomes which were reported by at least three studies. Finally, outcomes could not be studied according to AKI severity.

## Conclusions

Based on current knowledge, this meta-analysis identified in-hospital clinical outcomes associated with AKI in pediatric patients after cardiac surgery. CS-AKI is associated with a higher risk of mortality, cardiac arrhythmias, and the need for RRT, as well as longer ventilation time and PICU and hospital length of stay. Our findings highlight the need of establishing more adapted clinical protocols to reduce the disease burden of AKI after pediatric cardiac surgery. Furthermore, etiology-specific approaches to AKI are warranted, as outcomes are likely impacted by the underlying cause.

## Data Availability Statement

The original contributions presented in the study are included in the article/Supplementary Material, further inquiries can be directed to the corresponding author/s.

## Author Contributions

JV and HR: concept/design, data collection, data interpretation, drafting article, critical revision of article, and approval of article. MG, SK, KA, and DM: data interpretation, critical revision of article, and approval of article. All authors contributed to the article and approved the submitted version.

## Conflict of Interest

MG is proctor for Edwards and Medtronic. SK is consultant for GE Healthcare. The remaining authors declare that the research was conducted in the absence of any commercial or financial relationships that could be construed as a potential conflict of interest.

## Publisher's Note

All claims expressed in this article are solely those of the authors and do not necessarily represent those of their affiliated organizations, or those of the publisher, the editors and the reviewers. Any product that may be evaluated in this article, or claim that may be made by its manufacturer, is not guaranteed or endorsed by the publisher.

## Abbreviations

AKI: acute kidney injury
CI: confidence interval
CKD: chronic kidney disease
CPB: cardiopulmonary bypass
CS-AKI: cardiac surgery-associated acute kidney injury
MD: mean difference
MOOSE: Meta-analysis Of Observational Studies in Epidemiology
OR: odds ratio
PICU: pediatric intensive care unit
PRISMA: Preferred Reporting Items for Systematic reviews Meta-Analyses
RRT: renal replacement therapy.
