# Supplementary material for: In-Hospital Outcomes of Acute Kidney Injury After Pediatric Cardiac Surgery: A Meta-Analysis
Source: Front Pediatr. 2021 Sep 3;9:733744. doi: 10.3389/fped.2021.733744 (PMC8446539; doi:10.3389/fped.2021.733744)

**SUPPLEMENTARY MATERIAL**

“In-Hospital Outcomes of Acute Kidney Injury after Pediatric Cardiac Surgery:

A Meta-Analysis”

Jef Van den Eynde, BSc, Hajar Rotbi, Boris Delpire, BSc, Xander Jacquemyn, Ismat Pardi, Marc Gewillig, MD, PhD, Shelby Kutty, MD, PhD, MHCM, Karel Allegaert, MD, PhD, Djalila Mekahli, MD, PhD.

Supplementary Methods – *pages 2-3*

Supplementary Tables – *pages 4-12*

Supplementary Figures – *pages 13-16*

**Supplementary Methods**

***Search strategy.***

PubMed (n=1,656 on 6/08/2020)

(acute renal failure OR acute kidney failure OR acute renal injury OR acute kidney injury OR acute renal insufficiency OR AKI OR acute renal dysfunction OR acute kidney dysfunction) AND (cardiac OR heart) AND (surgery OR operation OR preoperative OR intraoperative OR perioperative) AND (pediatric OR neonate OR infant OR child OR adolescent) in all fields

Embase (n=1,275 on 6/08/2020)

('acute renal failure'/exp OR 'acute kidney failure'/exp OR 'acute renal injury'/exp OR 'acute kidney injury'/exp OR 'acute renal insufficiency'/exp OR 'aki' OR 'acute renal dysfunction' OR 'acute kidney dysfunction') AND ('cardiac' OR 'heart'/exp) AND ('surgery'/exp OR 'operation'/exp OR 'preoperative' OR 'intraoperative' OR 'perioperative') AND ('pediatric' OR 'neonate' OR 'infant' OR 'child' OR 'adolescent') in all fields

Scopus (n=1,256 on 6/08/2020)

( TITLE-ABS-KEY ( "acute renal failure" OR "acute kidney failure" OR "acute renal injury" OR "acute kidney injury" OR "acute renal insufficiency" OR "aki" OR "acute renal dysfunction" OR "acute kidney dysfunction" ) AND TITLE-ABS-KEY ( "cardiac" OR "heart" ) AND TITLE-ABS-KEY ( "surgery" OR "operation" OR "preoperative" OR "intraoperative" OR "perioperative" ) AND TITLE-ABS-KEY ( "pediatric" OR "neonate" OR "infant" OR "child" OR "adolescent" ) )

***Definitions of acute kidney injury (AKI)***

Three main definitions for AKI currently exist. First, the Risk for renal dysfunction, Injury to the kidney, Failure of kidney function, Loss of kidney function and End-stage renal disease (RIFLE) classification was published by the Acute Dialysis Quality Initiative in 2004 (1). Staging in this classification is based on changes from baseline serum creatinine (SCr) or glomerular filtration rate (GFR) within 7 days. This classification has been modified for children in the pediatric RIFLE (pRIFLE) classification, adding estimated creatinine clearance (eCrCl) (2). Secondly, another modification of the RIFLE classification has led to the AKI Network (AKIN) criteria, which focus on dynamic changes in creatinine (3). Contrary to RIFLE, AKIN does not use premorbid baseline SCr, but the lowest SCr within a 48-hour period, as the reference SCr for calculations of absolute and relative increase in SCr values. The AKIN criteria also avoid the use of CrCl. The third and last modification is the Kidney Disease Improving Global Outcomes (KDIGO) classification, which covers both the AKIN and RIFLE criteria (4). This new classification combines the 1.5-fold relative increase in SCr and urine output over 7 days from the RIFLE criteria with the absolute increase in SCr of 0.30 mg/dL over the rolling 48-hour window from AKIN. Urine output criteria are common to all three classifications.

**References**

1. Bellomo R, Ronco C, Kellum JA, Mehta RL, Palevsky P. Acute renal failure - definition, outcome measures, animal models, fluid therapy and information technology needs: the Second International Consensus Conference of the Acute Dialysis Quality Initiative (ADQI) Group. *Crit Care* (2004) 8(4):R204-212.
2. Akcan-Arikan A, Zappitelli M, Loftis LL, Washburn KK, Jefferson LS, Goldstein SL. Modified RIFLE criteria in critically ill children with acute kidney injury. *Kidney Int* (2007) 71(10):1028-1035.
3. Mehta RL, Kellum JA, Shah SV, et al. Acute Kidney Injury Network: report of an initiative to improve outcomes in acute kidney injury. *Crit Care* (2007) 11(2):R31.
4. Khwaja A. KDIGO clinical practice guidelines for acute kidney injury. *Nephron Clin Pract* (2012) 120(4):c179-184.

**Supplementary Tables**

**Table S1. Study characteristics.**

| **Study** | **Country of origin** | **Study design** | **Years of enrollment** | **Sample size** | **AKI incidence** | **Definition of AKI** | **Mean age (months)** |
| --- | --- | --- | --- | --- | --- | --- | --- |
| Pedersen 2008^1^ | Denmark | NP, NR, NM | 1993-2002 | 1128 | 11.5% | Defined as need for dialysis after surgery | 13.9 |
| Manrique 2009^2^ | USA | NP, NR, NM | 2004-2006 | 395 | 20.8% | pRIFLE | 21.8 |
| Zappitelli 2009^3^ | Canada | NP, NR, NM | 2002-2007 | 390 | 35.9% | pRIFLE | 33.5 |
| Krawczeski 2010^4^ | USA | NP, NR, NM | 2004-2007 | 374 | 31.8% | pRIFLE | 41.7 |
| Chiravuri 2011^5^ | USA | NP, NR, NM | 1998-2006 | 469 | 34.3% | pRIFLE | 33.9 |
| Jiao 2011^6^ | China | NP, NR, NM | 2008-2011 | 124 | 40.3% | AKIN | 5.1 |
| Krawczeski 2011^7^ | USA | NP, NR, NM | 2004-2007 | 220 | 27.3% | pRIFLE | 31.4 |
| Li 2011^8^ | Canada/USA | P, NR, M | 2007-2009 | 311 | 41.8% | AKIN | 45.2 |
| Moffett 2011^9^ | USA | NP, NR, NM | 2006-2007 | 415 | 21.2% | pRIFLE | 55.5 |
| Parikh 2011^10^ | Canada/USA | P, NR, M | 2007-2009 | 311 | 17.0% | Combination of pRIFLE and AKIN | 3.8 |
| Sethi 2011^11^ | India | NP, NR, NM | 2007-2009 | 124 | 11.3% | AKIN | 120.3 |
| Aydin 2012^12^ | USA | NP, NR, M | 2006-2009 | 692 | 33.8% | pRIFLE | 24.5 |
| Blinder 2012^13^ | USA | NP, NR, NM | 2003-2008 | 430 | 52.3% | Modified version of AKIN | 0.3 |
| Cantinotti 2012^14^ | Italy | P, NR, NM | 2010-2011 | 135 | 38.5% | pRIFLE | 17.0 |
| Hassinger 2012^15^ | USA | P, NR, NM | 2009-2010 | 100 | 28.0% | pRIFLE | 15.2 |
| Tóth 2012^16^ | Hungary | NP, NR, NM | 2004-2008 | 1510 | 31.9% | pRIFLE | 21.5 |
| AlAbbas 2013^17^ | Canada | NP, NR, NM | 2006-2009 | 122 | 62.3% | AKIN | 0.3 |
| Basu 2013^18^ | USA | NP, NR, NM | 1997-2008 | 92 | 19.6% | KDIGO | 0.2 |
| dos Santos El Halal 2013^19^ | Brazil | P, NR, NM | 2011-2012 | 85 | 44.7% | pRIFLE | 23.0 |
| Morgan 2013^20^ | Canada | NP, NR, M | 2002-2009 | 264 | 64.4% | AKIN | 0.6 |
| Morgan 2013 (2)^21^ | Canada | P, NR, NM | 2009-2010 | 109 | 69.7% | pRIFLE | 36.7 |
| Peco-Antic 2013^22^ | Serbia | P, NR, NM | 2011-2011 | 112 | 16.1% | pRIFLE | 24.6 |
| Ricci 2013^23^ | Italy | P, NR, NM | 2010-2011 | 160 | 56.3% | pRIFLE | 52.3 |
| Taylor 2013^24^ | USA | NP, NR, NM | 2009-2009 | 693 | 15.0% | Combination of pRIFLE and AKIN | 20.4 |
| Gil-Ruiz Gil-Esparza 2014^25^ | Spain | NP, NR, NM | 2008-2010 | 399 | 24.3% | pRIFLE | 23.3 |
| Jang 2014^26^ | South Korea | NP, NR, NM | 2003-2012 | 114 | 36.8% | pRIFLE | 7.6 |
| Lex 2014^27^ | Hungary | NP, NR, NM | 2004-2008 | 1489 | 27.8% | AKIN, pRIFLE, KDIGO | 21.7 |
| Esch 2015^28^ | USA | NP, NR, NM | 2003-2009 | 211 | 42.2% | AKIN | 2.7 |
| Piggott 2015^29^ | USA | NP, NR, NM | 2010-2013 | 95 | 45.3% | AKIN | 0.4 |
| Sethi 2015^30^ | India | NP, NR, NM | 2012-2013 | 208 | 9.6% | AKIN | 66.9 |
| Soni 2015^31^ | USA | NP, NR, NM | 2010-2012 | 778 | 41.3% | AKIN, pRIFLE | 23.9 |
| Zappitelli 2015^32^ | Canada/USA | P, NR, NM | 2007-2009 | 205 | 24.9% | KDIGO | 48.9 |
| Greenberg 2016^33^ | Canada/USA | P, NR, M | 2007-2009 | 131 | 44% | AKIN | 32.1 |
| Hollander 2016^34^ | USA | NP, NR, NM | 2007-2013 | 88 | 71.6% | KDIGO | 91.7 |
| Kumar 2016^35^ | USA | NP, NR, NM | 2010-2012 | 102 | 9.8% | AKIN | 0.8 |
| Park 2016^36^ | Korea | NP, NR, NM | 2012-2012 | 220 | 41.8% | KDIGO | 8.6 |
| Sugimoto 2016^37^ | Japan | P, NR, NM | 2010-2012 | 376 | 64.6% | pRIFLE | 25.4 |
| Wong 2016^38^ | USA | NP, NR, NM | 2007-2012 | 303 | 14.3% | KDIGO | 10.9 |
| Algaze 2017^39^ | USA | NP, NR, NM | 2004-2012 | 138 | 39.9% | KDIGO | 46.2 |
| Amini 2017^40^ | Iran | P, NR, NM | 2013-2016 | 519 | 28.9% | pRIFLE | 28.3 |
| Blinder 2017^41^ | USA | NP, R, M | 2006-2012 | 799 | 36.2% | AKIN | 4.4 |
| Hirano 2017^42^ | Japan | NP, NR, NM | 2007-2013 | 418 | 24.9% | pRIFLE | 91.2 |
| Kim-Campbell 2017^43^ | USA | P, NR, NM | 2012-2016 | 60 | 56.7% | KDIGO | 50.7 |
| Lee 2017^44^ | Korea | NP, NR, NM | 2013-2013 | 135 | 14.1% | pRIFLE | 51.8 |
| Patterson 2017^45^ | USA | NP, NR, NM | 2005-2012 | 186 | 52.2% | Combination of pRIFLE and AKIN | 37.8 |
| Reyes-Flandes 2017^46^ | Mexico | NP, NR, NM | 2014-2015 | 91 | 34.1% | pRIFLE | 20.0 |
| Tanyildiz 2017^47^ | Turkey | NP, NR, NM | 2009-2011 | 137 | 58.2% | AKIN, pRIFLE | 43.0 |
| Greenberg 2018^48^ | Canada/USA | P, NR, M | 2007-2009 | 110 | 44.5% | Defined as a doubling in serum creatinine concentration from baseline or receiving acute dialysis during hospital stay | 44.8 |
| Hu 2018^49^ | China | NP, NR, NM | 2013-2016 | 1026 | 11.5% | AKIN | 36.6 |
| Lee 2018^50^ | Korea | NP, NR, NM | 2013-2014 | 505 | 36.6% | KDIGO | 10.2 |
| SooHoo 2018^51^ | USA | NP, NR, NM | 2009-2015 | 95 | 40.0% | KDIGO | 0.2 |
| Graziani 2019^52^ | Argentina | NP, NR, NM | 2015-2017 | 125 | 35.2% | KDIGO | 9.0 |
| Ueno 2019^53^ | Japan | NP, NR, NM | 2013-2016 | 145 | 37.9% | KDIGO | 4.9 |
| Wang 2019^54^ | China | NP, NR, NM | 2017-2017 | 89 | 67.4% | pRIFLE | 17.4 |
| Baek 2020^55^ | Korea | NP, NR, NM | 2017-2018 | 30 | 40.0% | KDIGO | 2.9 |
| Huynh 2020^56^ | Canada | P, NR, M | 2005-2012 | 58 | 57% | KDIGO | 0.32 |
| Ueno 2020^57^ | Japan | NP, NR, NM | 2010-2018 | 81 | 70.4% | KDIGO | 0.5 |
| Yoneyama 2020^58^ | Japan | P, NR, NM | 2017-2018 | 103 | 45.6% | KDIGO | 54.3 |
| AKI, acute kidney injury; AKIN, Acute Kidney Injury Network; KDIGO, Kidney Disease Improving Global Outcomes; M, multicenter; NM, single-center; NP, retrospective; NR, non-randomized; P, prospective; pRIFLE, pediatric RIFLE; R, randomized; RIFLE, Risk for renal dysfunction, Injury to the kidney, Failure of kidney function, Loss of kidney function and End-stage renal disease. | | | | | | | |

**References**

1. Pedersen KR, Hjortdal VE, Christensen S, et al. Clinical outcome in children with acute renal failure treated with peritoneal dialysis after surgery for congenital heart disease. *Kidney Int Suppl.* 2008(108):S81-86.

2. Manrique A, Jooste EH, Kuch BA, et al. The association of renal dysfunction and the use of aprotinin in patients undergoing congenital cardiac surgery requiring cardiopulmonary bypass. *Anesth Analg.* 2009;109(1):45-52.

3. Zappitelli M, Bernier PL, Saczkowski RS, et al. A small post-operative rise in serum creatinine predicts acute kidney injury in children undergoing cardiac surgery. *Kidney Int.* 2009;76(8):885-892.

4. Krawczeski CD, Vandevoorde RG, Kathman T, et al. Serum cystatin C is an early predictive biomarker of acute kidney injury after pediatric cardiopulmonary bypass. *Clin J Am Soc Nephrol.* 2010;5(9):1552-1557.

5. Chiravuri SD, Riegger LQ, Christensen R, et al. Factors associated with acute kidney injury or failure in children undergoing cardiopulmonary bypass: a case-controlled study. *Paediatr Anaesth.* 2011;21(8):880-886.

6. Jiao YQ, Zhou GX, Huang JP, Hong XY, Yang XY, Feng ZC. Acute Kidney Injury in Children Undergoing Correction of Congenital Heart Disease. *Applied Mechanics and Materials.* 2011;140:84-90.

7. Krawczeski CD, Goldstein SL, Woo JG, et al. Temporal relationship and predictive value of urinary acute kidney injury biomarkers after pediatric cardiopulmonary bypass. *J Am Coll Cardiol.* 2011;58(22):2301-2309.

8. Li S, Krawczeski CD, Zappitelli M, et al. Incidence, risk factors, and outcomes of acute kidney injury after pediatric cardiac surgery: a prospective multicenter study. *Crit Care Med.* 2011;39(6):1493-1499.

9. Moffett BS, Goldstein SL, Adusei M, Kuzin J, Mohan P, Mott AR. Risk factors for postoperative acute kidney injury in pediatric cardiac surgery patients receiving angiotensin-converting enzyme inhibitors. *Pediatr Crit Care Med.* 2011;12(5):555-559.

10. Parikh CR, Devarajan P, Zappitelli M, et al. Postoperative biomarkers predict acute kidney injury and poor outcomes after pediatric cardiac surgery. *J Am Soc Nephrol.* 2011;22(9):1737-1747.

11. Sethi SK, Goyal D, Yadav DK, et al. Predictors of acute kidney injury post-cardiopulmonary bypass in children. *Clin Exp Nephrol.* 2011;15(4):529-534.

12. Aydin SI, Seiden HS, Blaufox AD, et al. Acute kidney injury after surgery for congenital heart disease. *Ann Thorac Surg.* 2012;94(5):1589-1595.

13. Blinder JJ, Goldstein SL, Lee VV, et al. Congenital heart surgery in infants: effects of acute kidney injury on outcomes. *J Thorac Cardiovasc Surg.* 2012;143(2):368-374.

14. Cantinotti M, Storti S, Lorenzoni V, et al. The combined use of neutrophil gelatinase-associated lipocalin and brain natriuretic peptide improves risk stratification in pediatric cardiac surgery. *Clin Chem Lab Med.* 2012;50(11):2009-2017.

15. Hassinger AB, Backer CL, Lane JC, Haymond S, Wang D, Wald EL. Predictive power of serum cystatin C to detect acute kidney injury and pediatric-modified RIFLE class in children undergoing cardiac surgery. *Pediatr Crit Care Med.* 2012;13(4):435-440.

16. Toth R, Breuer T, Cserep Z, et al. Acute kidney injury is associated with higher morbidity and resource utilization in pediatric patients undergoing heart surgery. *Ann Thorac Surg.* 2012;93(6):1984-1990.

17. Alabbas A, Campbell A, Skippen P, Human D, Matsell D, Mammen C. Epidemiology of cardiac surgery-associated acute kidney injury in neonates: a retrospective study. *Pediatr Nephrol.* 2013;28(7):1127-1134.

18. Basu RK, Andrews A, Krawczeski C, Manning P, Wheeler DS, Goldstein SL. Acute kidney injury based on corrected serum creatinine is associated with increased morbidity in children following the arterial switch operation. *Pediatr Crit Care Med.* 2013;14(5):e218-224.

19. dos Santos El Halal MG, Carvalho PR. Acute kidney injury according to pediatric RIFLE criteria is associated with negative outcomes after heart surgery in children. *Pediatr Nephrol.* 2013;28(8):1307-1314.

20. Morgan CJ, Zappitelli M, Robertson CM, et al. Risk factors for and outcomes of acute kidney injury in neonates undergoing complex cardiac surgery. *J Pediatr.* 2013;162(1):120-127 e121.

21. Morgan CJ, Gill PJ, Lam S, Joffe AR. Peri-operative interventions, but not inflammatory mediators, increase risk of acute kidney injury after cardiac surgery: a prospective cohort study. *Intensive Care Med.* 2013;39(5):934-941.

22. Peco-Antic A, Ivanisevic I, Vulicevic I, et al. Biomarkers of acute kidney injury in pediatric cardiac surgery. *Clin Biochem.* 2013;46(13-14):1244-1251.

23. Ricci Z, Di Nardo M, Iacoella C, Netto R, Picca S, Cogo P. Pediatric RIFLE for acute kidney injury diagnosis and prognosis for children undergoing cardiac surgery: a single-center prospective observational study. *Pediatr Cardiol.* 2013;34(6):1404-1408.

24. Taylor ML, Carmona F, Thiagarajan RR, et al. Mild postoperative acute kidney injury and outcomes after surgery for congenital heart disease. *J Thorac Cardiovasc Surg.* 2013;146(1):146-152.

25. Gil-Ruiz Gil-Esparza MA, Alcaraz Romero AJ, Romero Otero A, et al. Prognostic relevance of early AKI according to pRIFLE criteria in children undergoing cardiac surgery. *Pediatr Nephrol.* 2014;29(7):1265-1272.

26. Jang WS, Kim WH, Choi K, et al. Incidence, risk factors and clinical outcomes for acute kidney injury after aortic arch repair in paediatric patients. *Eur J Cardiothorac Surg.* 2014;45(6):e208-214.

27. Lex DJ, Toth R, Cserep Z, et al. A comparison of the systems for the identification of postoperative acute kidney injury in pediatric cardiac patients. *Ann Thorac Surg.* 2014;97(1):202-210.

28. Esch JJ, Salvin JM, Thiagarajan RR, Del Nido PJ, Rajagopal SK. Acute kidney injury after Fontan completion: Risk factors and outcomes. *J Thorac Cardiovasc Surg.* 2015;150(1):190-197.

29. Piggott KD, Soni M, Decampli WM, et al. Acute Kidney Injury and Fluid Overload in Neonates Following Surgery for Congenital Heart Disease. *World J Pediatr Congenit Heart Surg.* 2015;6(3):401-406.

30. Sethi SK, Kumar M, Sharma R, Bazaz S, Kher V. Acute Kidney Injury in Children After Cardiopulmonary Bypass: Risk Factors and Outcome. *Indian Pediatrics.* 2015;52:223-226.

31. Soni M, Piggott KD, DeCampli W, et al. Are We Overdiagnosing Acute Kidney Injury in Pediatric Patients Following Cardiac Surgery? *World J Pediatr Congenit Heart Surg.* 2015;6(4):496-501.

32. Zappitelli M, Greenberg JH, Coca SG, et al. Association of definition of acute kidney injury by cystatin C rise with biomarkers and clinical outcomes in children undergoing cardiac surgery. *JAMA Pediatr.* 2015;169(6):583-591.

33. Greenberg JH, Zappitelli M, Devarajan P, et al. Kidney Outcomes 5 Years After Pediatric Cardiac Surgery: The TRIBE-AKI Study. *JAMA Pediatr.* 2016;170(11):1071-1078.

34. Hollander SA, Montez-Rath ME, Axelrod DM, et al. Recovery From Acute Kidney Injury and CKD Following Heart Transplantation in Children, Adolescents, and Young Adults: A Retrospective Cohort Study. *Am J Kidney Dis.* 2016;68(2):212-218.

35. Kumar TK, Allen Ccp J, Spentzas Md T, et al. Acute Kidney Injury Following Cardiac Surgery in Neonates and Young Infants: Experience of a Single Center Using Novel Perioperative Strategies. *World J Pediatr Congenit Heart Surg.* 2016;7(4):460-466.

36. Park SK, Hur M, Kim E, et al. Risk Factors for Acute Kidney Injury after Congenital Cardiac Surgery in Infants and Children: A Retrospective Observational Study. *PLoS One.* 2016;11(11):e0166328.

37. Sugimoto K, Toda Y, Iwasaki T, et al. Urinary Albumin Levels Predict Development of Acute Kidney Injury After Pediatric Cardiac Surgery: A Prospective Observational Study. *J Cardiothorac Vasc Anesth.* 2016;30(1):64-68.

38. Wong JH, Selewski DT, Yu S, et al. Severe Acute Kidney Injury Following Stage 1 Norwood Palliation: Effect on Outcomes and Risk of Severe Acute Kidney Injury at Subsequent Surgical Stages. *Pediatr Crit Care Med.* 2016;17(7):615-623.

39. Algaze CA, Koth AM, Faberowski LW, Hanley FL, Krawczeski CD, Axelrod DM. Acute Kidney Injury in Patients Undergoing the Extracardiac Fontan Operation With and Without the Use of Cardiopulmonary Bypass. *Pediatr Crit Care Med.* 2017;18(1):34-43.

40. Amini S, Abbaspour H, Morovatdar N, Robabi HN, Soltani G, Tashnizi MA. Risk Factors and Outcome of Acute Kidney Injury after Congenital Heart Surgery: A Prospective Observational Study. *Indian J Crit Care Med.* 2017;21(12):847-851.

41. Blinder JJ, Asaro LA, Wypij D, et al. Acute Kidney Injury After Pediatric Cardiac Surgery: A Secondary Analysis of the Safe Pediatric Euglycemia After Cardiac Surgery Trial. *Pediatr Crit Care Med.* 2017;18(7):638-646.

42. Hirano D, Ito A, Yamada A, et al. Independent Risk Factors and 2-Year Outcomes of Acute Kidney Injury after Surgery for Congenital Heart Disease. *Am J Nephrol.* 2017;46(3):204-209.

43. Kim-Campbell N, Gretchen C, Callaway C, et al. Cell-Free Plasma Hemoglobin and Male Gender Are Risk Factors for Acute Kidney Injury in Low Risk Children Undergoing Cardiopulmonary Bypass. *Crit Care Med.* 2017;45(11):e1123-e1130.

44. Lee SH, Kim SJ, Kim HJ, Son JS, Lee R, Yoon TG. Acute Kidney Injury Following Cardiopulmonary Bypass in Children- Risk Factors and Outcomes. *Circ J.* 2017;81(10):1522-1527.

45. Patterson T, Hehir DA, Buelow M, et al. Hemodynamic Profile of Acute Kidney Injury Following the Fontan Procedure: Impact of Renal Perfusion Pressure. *World J Pediatr Congenit Heart Surg.* 2017;8(3):367-375.

46. Reyes-Flandes EN, Herrera-Landero A, Bobadilla-Gonzalez P, Nunez-Enriquez JC. [Risk factors associated with postoperative acute renal failure in pediatric patients undergoing cardiopulmonary bypass surgery]. *Rev Chil Pediatr.* 2017;88(2):209-215.

47. Tanyildiz M, Ekim M, Kendirli T, et al. Acute kidney injury in congenital cardiac surgery: Pediatric risk-injury-failure-loss-end-stage renal disease and Acute Kidney Injury Network. *Pediatr Int.* 2017;59(12):1252-1260.

48. Greenberg JH, Devarajan P, Thiessen-Philbrook HR, et al. Kidney injury biomarkers 5 years after AKI due to pediatric cardiac surgery. *Pediatr Nephrol.* 2018;33(6):1069-1077.

49. Hu GH, Duan L, Jiang M, Zhang CL, Duan YY. Wider intraoperative glycemic fluctuation increases risk of acute kidney injury after pediatric cardiac surgery. *Ren Fail.* 2018;40(1):611-617.

50. Lee JH, Jung JY, Park SW, et al. Risk factors of acute kidney injury in children after cardiac surgery. *Acta Anaesthesiol Scand.* 2018;62(10):1374-1382.

51. SooHoo MM, Patel SS, Jaggers J, Faubel S, Gist KM. Acute Kidney Injury Defined by Fluid Corrected Creatinine in Neonates After the Norwood Procedure. *World J Pediatr Congenit Heart Surg.* 2018;9(5):513-521.

52. Graziani MP, Moser M, Bozzola CM, et al. Acute kidney injury in children after cardiac surgery: Risk factors and outcomes. A retrospective, cohort study. *Arch Argent Pediatr.* 2019;117(6):e557-e567.

53. Ueno K, Seki S, Shiokawa N, et al. Validation of acute kidney injury according to the modified KDIGO criteria in infants after cardiac surgery for congenital heart disease. *Nephrology (Carlton).* 2019;24(3):294-300.

54. Wang C, Fu P, Wang Y, et al. Epidemiology of acute kidney injury among paediatric patients after repair of anomalous origin of the left coronary artery from the pulmonary artery. *Eur J Cardiothorac Surg.* 2019;56(5):883-890.

55. Baek HS, Lee Y, Jang HM, et al. Variation in clinical usefulness of biomarkers of acute kidney injury in young children undergoing cardiac surgery. *Clin Exp Pediatr.* 2020;63(4):151-156.

56. Huynh L, Rodriguez-Lopez S, Benisty K, et al. Follow-up after neonatal heart disease repair: watch out for chronic kidney disease and hypertension! *Pediatr Nephrol.* 2020;35(11):2137-2145.

57. Ueno K, Shiokawa N, Takahashi Y, et al. Kidney Disease: Improving Global Outcomes in neonates with acute kidney injury after cardiac surgery. *Clin Exp Nephrol.* 2020;24(2):167-173.

58. Yoneyama F, Okamura T, Takigiku K, Yasukouchi S. Novel Urinary Biomarkers for Acute Kidney Injury and Prediction of Clinical Outcomes After Pediatric Cardiac Surgery. *Pediatr Cardiol.* 2020;41(4):695-702.

**Supplementary Figures**

**Figure S1. Risk of bias assessment of observational studies using the ROBINS-I tool (1).**

**
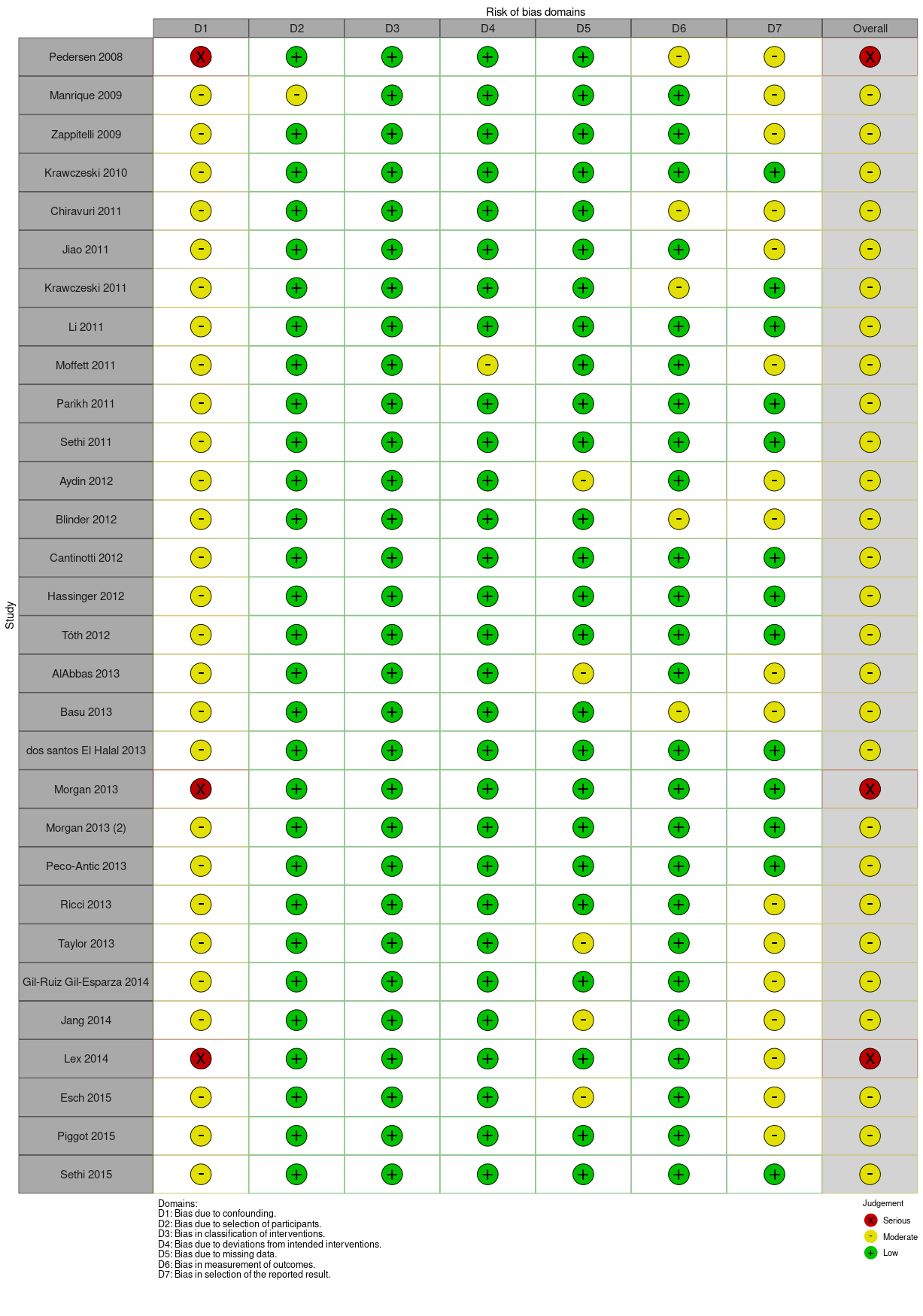
**

**Figure S2. Risk of bias assessment of observational studies using the ROBINS-I tool (2).**


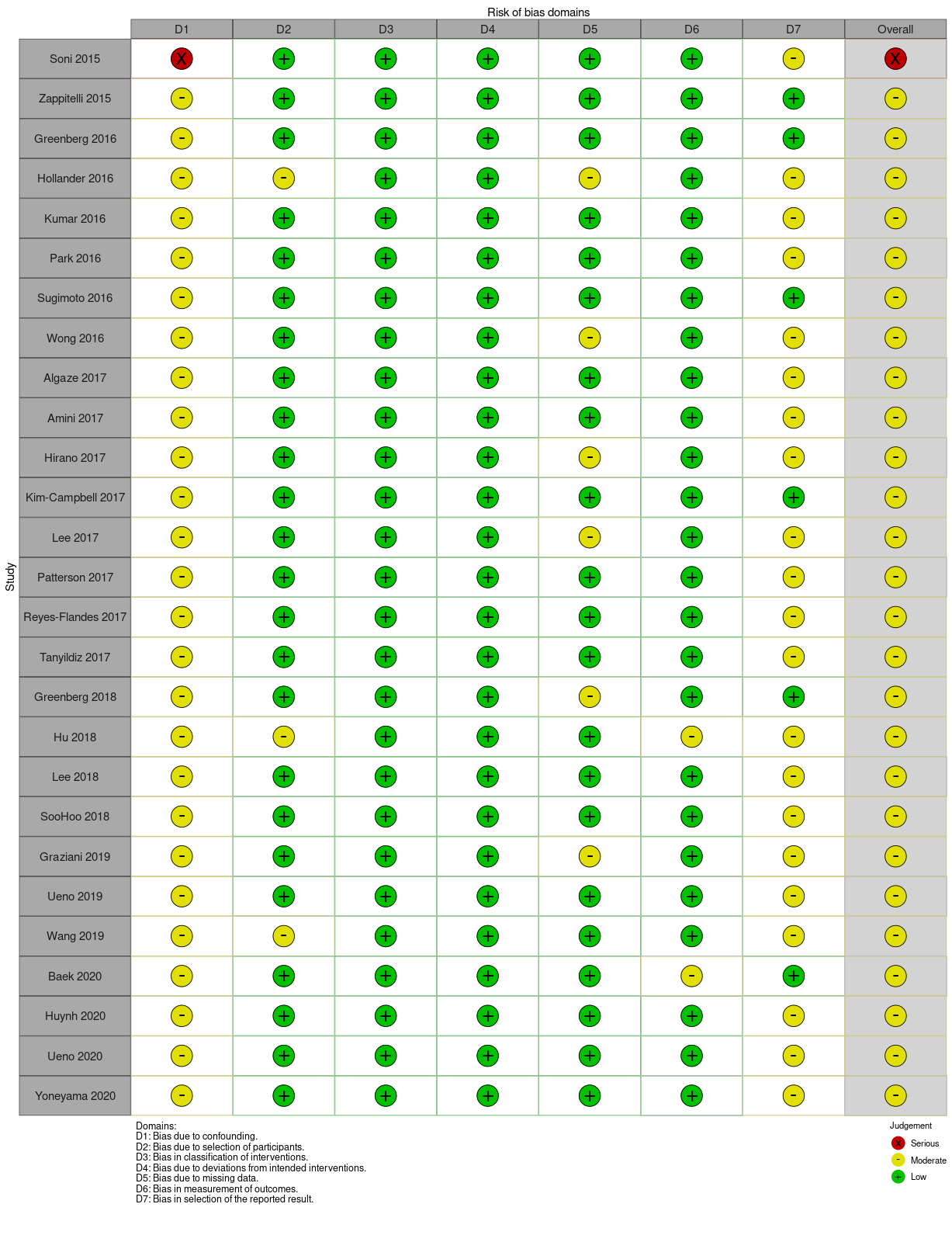


**Figure S3. Risk of bias assessment of randomized controlled trials using the RoB 2 tool.**

**
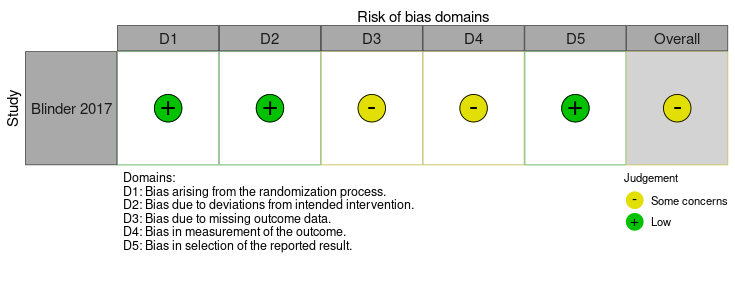
**

**Figure S4. Publication bias analysis by funnel plot graphic: in-hospital outcomes.**

(A) in-hospital mortality, (B) need for RRT, (C) cardiac arrhythmias, (D) ventilation time (days), (E) PICU length of stay, and (F) hospital length of stay. Funnel plot analysis disclosed asymmetry around the axis for need for RRT (Egger’s p<0.001, Begg and Mazumdar’s p=0.777) and ventilation time (Egger’s p=0.022, Begg and Mazumdar’s p=0.870). PICU = pediatric intensive care unit; RRT = renal replacement therapy.


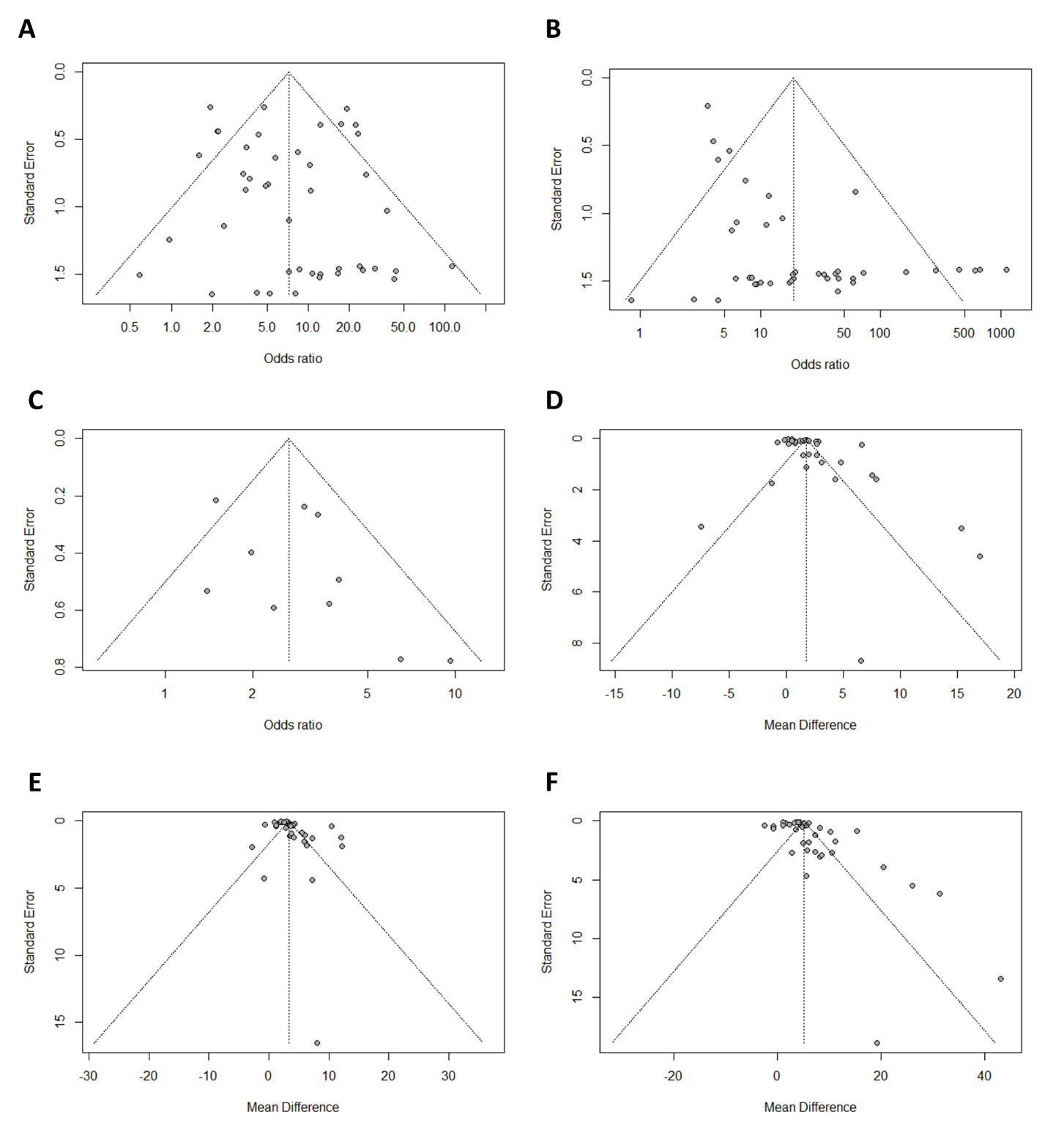

Supplement: Supplementary file 1 [file Data_Sheet_1.docx]
